# Characterization of novel markers of senescence and their prognostic potential in cancer

**DOI:** 10.1038/cddis.2014.489

**Published:** 2014-11-20

**Authors:** M Althubiti, L Lezina, S Carrera, R Jukes-Jones, S M Giblett, A Antonov, N Barlev, G S Saldanha, C A Pritchard, K Cain, S Macip

**Affiliations:** 1 Department of Biochemistry, University of Leicester, Leicester, UK; 2 Department of Biochemistry, Faculty of Medicine, Umm AL-Qura University, Mecca, Saudi Arabia; 3 Institute of Cytology RAS, Saint-Petersburg, Russia; 4 MRC Toxicology Unit, Leicester, UK; 5 Department of Cancer Studies and Molecular Medicine, University of Leicester, Leicester, UK

## Abstract

Cellular senescence is a terminal differentiation state that has been proposed to have a role in both tumour suppression and ageing. This view is supported by the fact that accumulation of senescent cells can be observed in response to oncogenic stress as well as a result of normal organismal ageing. Thus, identifying senescent cells in *in vivo* and *in vitro* has an important diagnostic and therapeutic potential. The molecular pathways involved in triggering and/or maintaining the senescent phenotype are not fully understood. As a consequence, the markers currently utilized to detect senescent cells are limited and lack specificity. In order to address this issue, we screened for plasma membrane-associated proteins that are preferentially expressed in senescent cells. We identified 107 proteins that could be potential markers of senescence and validated 10 of them (DEP1, NTAL, EBP50, STX4, VAMP3, ARMX3, B2MG, LANCL1, VPS26A and PLD3). We demonstrated that a combination of these proteins can be used to specifically recognize senescent cells in culture and in tissue samples and we developed a straightforward fluorescence-activated cell sorting-based detection approach using two of them (DEP1 and B2MG). Of note, we found that expression of several of these markers correlated with increased survival in different tumours, especially in breast cancer. Thus, our results could facilitate the study of senescence, define potential new effectors and modulators of this cellular mechanism and provide potential diagnostic and prognostic tools to be used clinically.

Apoptosis and senescence are the two main processes that prevent the emergence of transformed cells.^1^ Senescence is usually defined as a permanent cell cycle arrest in which cells remain metabolically active and adopt characteristic phenotypic changes.^2^ Senescent cells often appear multinucleated, large and extended, and exhibit spindle and vacuolization features.^3^ The onset of this phenotype is believed to be either the result of telomere shortening after a number of cell divisions (replicative senescence) or a response to a diverse range of stress stimuli (stress-induced premature senescence).^3, 4^

Expression of oncogenes, such as Ras, cyclin E, E2F3 and Raf, can also trigger senescence, underscoring its tumour-suppressing properties.^5, 6, 7^ Moreover, presence of senescent cells *in vivo* is often observed in the pre-malignant stages of a tumour; they gradually disappear, suggesting that the senescent barrier needs to be overcome in order to progress into full malignancy.^8, 9, 10^ Cell senescence has also been associated with age-dependent organismal changes in rodents and primates,^11, 12, 13^ and accumulation of senescent cells has been shown to contribute to the functional impairment of different organs.^14^ This has led to the hypothesis that senescence is an antagonistically pleiotropic process, with beneficial effects in the early decades of life as a tumour suppressor but detrimental to fitness and survival in later stages, because of its contribution to age-related pathologies.^15^

Despite the considerable knowledge accumulated in the 50 years since Leonard Hayflick first described the phenomenon,^16^ the molecular pathways involved in senescence have not been fully characterized.^17^ One of the well-known features of both replicative and stress-induced senescence is the participation of the p53-p21 and/or p16-RB axis in the phenotype. Although *in vivo* suppression of p53 and/or its upstream regulator ARF is enough to prevent senescence in some models,^18^ other cell types rely primarily on p16 for its induction.^19^ The p53 target gene, p21, has often been considered critical for establishing senescence, whereas p16 may be more involved in the maintenance of the phenotype,^20^ an effect also achieved by an increase in intracellular reactive oxygen species.^21, 22^ Cellular senescence is associated with the secretion of growth factors, chemokines and cytokines, collectively known as the senescence-associated secretory phenotype (SASP). SASP has an effect on cell proliferation and angiogenesis, as well as a possible role in promoting aging and tumourigenesis.^23, 24^ It can also promote migration of leukocytes and tumour cells, which in turn may induce tumour metastasis.^25^

Increased expression of intracellular and/or secreted proteins, such as p21, p16, macroH2A, IL-6, phosphorylated p38 MAPK, PPP1A, Smurf2 or PGM,^26, 27, 28, 29, 30^ has been used as a surrogate marker of senescence, although it does not provide a specific measurement.^18^ Senescent cells display different modifications in the organization of chromatin that can help identify them as well. In normal cells, DNA staining reveals completely uniform colour outlines, whereas senescent cells usually show dot-like patterns, known as senescence-associated heterochromatic foci (SAHF), which appear because of intensive remodelling in the chromatin and a lower susceptibility for digestion by nucleases.^31, 32^ SAHF development is not necessary for the establishment of senescence and its presence depends on cell type and the triggering stimuli.^33^

Apart from these factors, the most distinctive measurable feature of senescent cells is the presence of a specific *β*-galactosidase enzymatic activity at pH 6.0, different from the normally observed at pH 4.0 within lysosomes.^34^ This has been named senescence-associated *β*-galactosidase (SA-*β*-Gal) and it is thought to be a consequence of the enlargement in the structures of lysosome in senescent cells, without having a known role in the establishment or maintenance of the phenotype.^35^ Although it is currently the standard for detecting senescent cells, several conditions, such as high cell confluence or treatment with hydrogen peroxide, can also stimulate SA-*β*-Gal activity, leading to many false positives.^36^ In summary, none of the currently available markers are sufficient on their own for conclusively identifying senescent cells *in vivo* or *in vitro*, which underscores the need for better characterization tools.^30^

Here, we describe and validate a list of novel senescent-specific proteins associated with the plasma membrane, uncovered through a proteomic screening, which define a profile that can easily be interrogated in a specific and quantitative manner using different techniques. We propose to use them as potential selective markers of senescence and we also anticipate that they may have a role as effectors and/or modulators, which would uncover novel pathways involved in the process. Moreover, we explored their prognostic potential and found a correlation between their expression and patient survival in different types of cancer, consistent of the role of senescence as an important tumour-suppressor mechanism.

## Results

### Proteomic analysis of the expression of proteins associated with the plasma membrane in senescent cells

**Figure 1:**
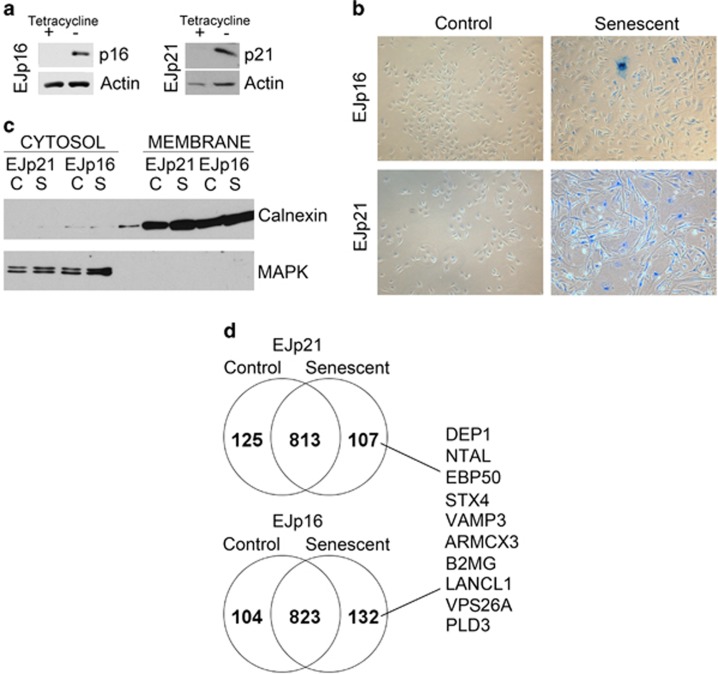
Analysis of the membrane faction of senescence EJp16 and EJp21. (**a**) Western blots of EJp16 and EJp21 without and with induced expression of exogenous p16 or p21, respectively, as determined by the presence of tet in the culture medium. (**b**) SA-*β*-Gal staining of EJp16 and EJp21 uninduced (Control) or 4 days after tet removal to induce the expression of exogenous p16 or p21 (Senescent). Blue staining and morphological changes are indicative of senescence. (**c**) Western blot analysis of lysates separated into cytosolic and membrane fractions of EJp21 and EJp16 uninduced (C) or 4 days after tet removal (S). Calnexin is used as a marker of membrane proteins and MAPK as a marker of the cytosolic fraction. (**d**) Number of membrane proteins differentially expressed in control and senescent EJp21 and EJp16, compared with those present in both conditions, together with a list of targets selected for validation, as determined by mass spectrometry

In order to characterize the profile of proteins selectively associated with the cell membrane after the induction of senescence, we used a bladder cancer cell line, EJ, with a tetracycline (tet)-regulatable p21 or p16 expression system (Figure 1a). These cells, named EJp21 and EJp16, respectively,^22, 37^ irreversibly senesce after prolonged expression of the induced protein (Figure 1b and Supplementary Figure 1A). We isolated the membrane fraction from lysates of these cells (Figure 1c) and performed a mass spectrometry screen comparing the senescent cells with their non-induced counterparts. As shown in Figure 1d, 107 proteins were exclusively present in membranes of senescent EJp21 and 132 in EJp16. Seventeen were present in both senescent cells but in none of the controls. Among these proteins, DCR2, NOTCH3 and ICAM1 were detected, all of which had been previously reported to be increased in senescence.^10, 38, 39^ This confirmed the suitability of the proteomics protocols used for our screen. We then selected 10 proteins from the analysis for further validation: DEP1, NTAL, EBP50, STX4, VAMP3, ARMCX3, B2MG, LANCL1, VPS26A and PLD3. They were chosen because none of them had been previously been associated with senescence and they were all predicted to be present on the plasma membrane using available algorithms (http://www.enzim.hu/hmmtop/html/submit.html).

### Validation of potential membrane markers of senescent cells

**Figure 2:**
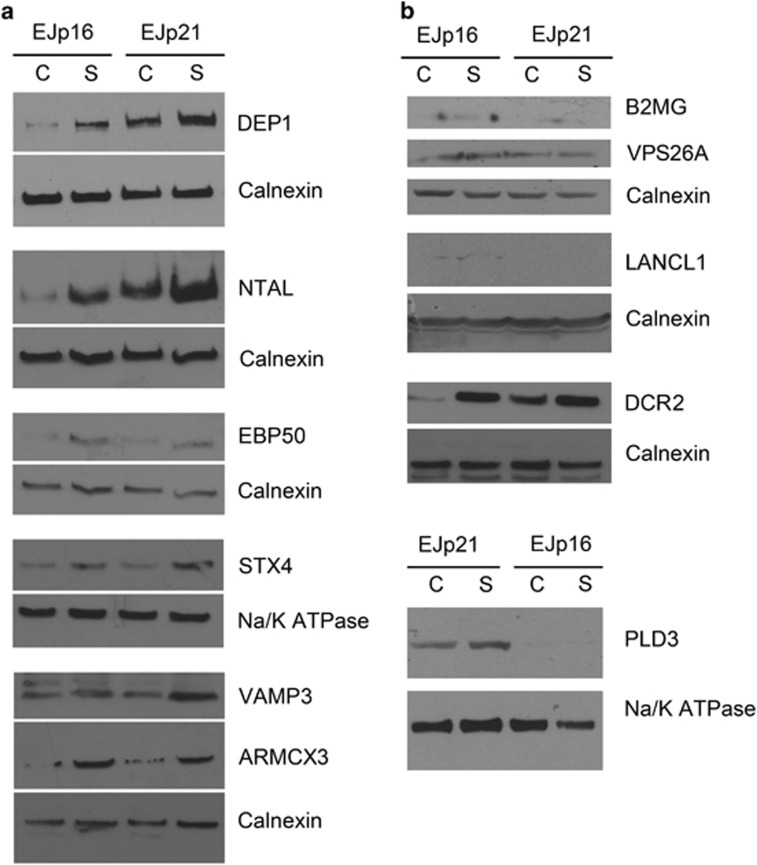
Western blot validation of senescent-specific targets in EJp16 and EJp21. (**a** and **b**) Protein expression of selected targets in the membrane fraction of lysates from EJp16 and EJp21 uninduced (C) or 4 days after tet removal (S). Calnexin and Na/K ATPase are used as membrane-specific loading controls

We next confirmed that the selected proteins were indeed expressed preferentially in the membranes of senescent cells. To this end, we used lysates with the cell membrane fraction from EJp16 and EJp21 induced to senesce. As shown in Figure 2a, basal levels of DEP1, NTAL, EBP50, STX4 VAMP3 and ARMCX3 were low in membrane lysates of uninduced EJp16 cells. After 5 days of p16 expression, when cells are known to be irreversibly senescent,^22^ expression of these proteins was significantly increased, except for VAMP3, which only show minor induction (Figure 2a and Supplementary Figure 1B). DEP1 and NTAL were notably expressed in EJp21 in basal conditions, and were slightly upregulated after 5 days of p21 induction. EBP50, STX4 and ARMCX3 displayed low basal levels of expression followed by a substantial increase after EJp21 entered senescence. VAMP3 only showed a small increase in induced EJp21 cells. As shown in Figure 2b (and Supplementary Figure 1B), B2MG, VPS26A and LANCL1 and PLD3 were not induced significantly in any senescent model. Finally, DCR2 was shown to be induced in both p16- and p21-dependent senescence, as expected,^10, 40^ although its increase was much higher in EJp16. The results were similar using whole-cell lysates and none of the markers tested were present in the parental EJ cell line (Supplementary Figure 2A). All these results together confirmed that six of the potential markers (DEP1, NTAL, EBP50, STX4, VAMP3 and ARMCX3) were upregulated in senescent cells, although at different levels, and three more (B2MG, LANCL1 and VPS26A) were not significantly induced, according to western blots. There were also p21- and p16-specific patterns of expression.

**Figure 3:**
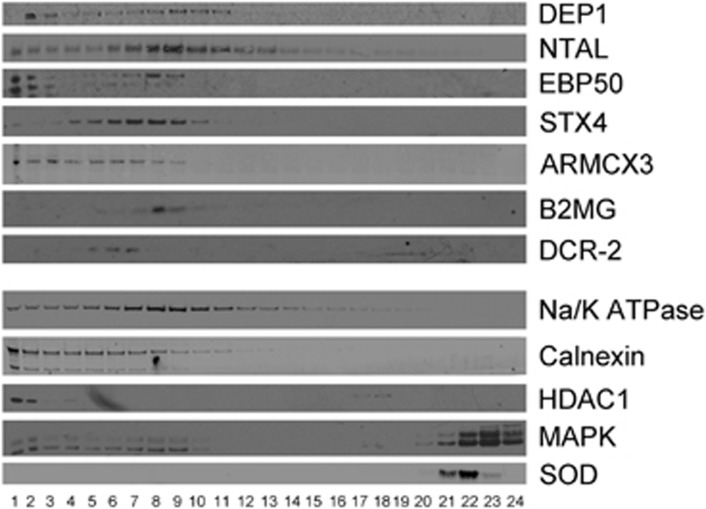
Expression of selected targets in membranes of senescent cells by cell fractionation. In all, 10–50% sucrose density gradient separation of lysates from EJp16, 4 days after tet removal. Calnexin and Na/K ATPase are used as markers of the cell membrane fractions. HDAC1 is used as marker of the nuclear fraction. MAPK is used as marker of the cytosolic fractions. SOD is used as marker of the mitochondrial fraction

**Figure 4:**
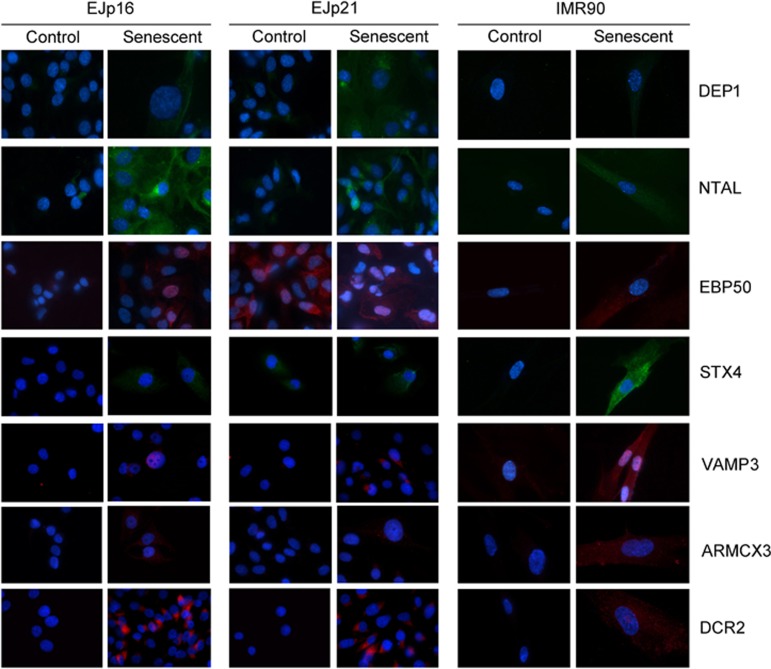
Expression and localization of senescence markers. Immunofluorescent images of selected targets in EJp16 and EJp21 uninduced (Control) or 4 days after tet removal (Senescent), as well as early passage IMR90 human fibroblasts compared with those entering replicative senescence after serial passaging. Nucleus are stained with DAPI (blue)

We further validated these results using fractionation by sucrose gradient of whole-cell lysates of senescent EJp16. Figure 3 shows that DEP1, NTAL, EBP50, STX4 and ARMCX3 colocalize in the same fraction as cell membrane markers Na/K ATPase and Calnexin. B2MG shows low levels of expression, consistent with Figure 2. This supports the localization of these proteins in the plasma membrane. We also used immunofluorescence microscopy to study expression of these proteins (Figure 4). DEP1, NTAL, EBP50 and STX4 showed induction in senescent EJp16, similar to the positive control, DCR2. VAMP3 and ARMCX3 also showed upregulation, but at lower levels. In EJp21, all markers were significantly increased, except STX4, which only showed a moderate elevation, and EBP50. The expression of these proteins in IMR90 human fibroblasts was also measured, comparing early passage cells with those induced to senesce after serial passaging (see SA-*β*-Gal staining in Supplementary Figure 1C) or in normal diploid fibroblasts after ras-mediated oncogene-induced senescence. All the proteins tested showed low basal levels in dividing fibroblasts and increased expression in senescent ones (Figure 4 and Supplementary Figure 2B), confirming that they could also be used as markers of replicative senescence in normal cells.

### Defining a protocol for rapid detection of senescence cells by fluorescence-activated cell sorting (FACS) analysis

**Figure 5:**
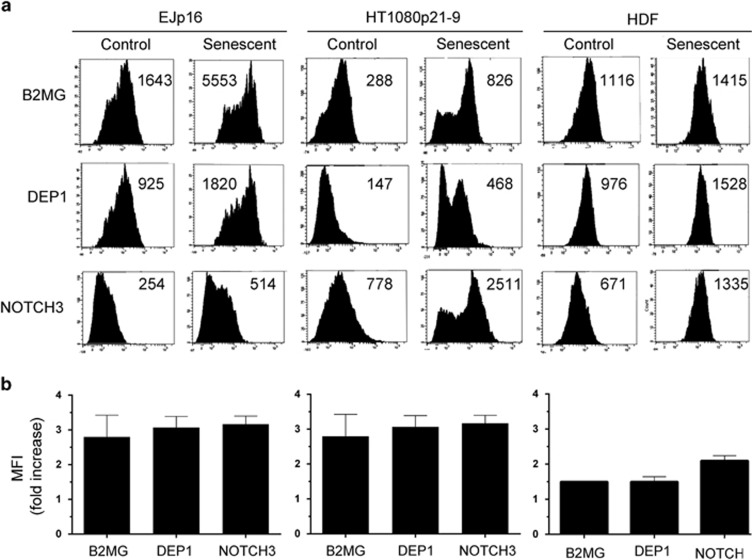
Defining a new FACS-based protocol for the detection of senescent cells. (**a**) Representative plot analysis of fluorescence levels in control and senescent EJp16, HT1080p21-9 and human diploid fibroblasts (HDF) stained with fluorescently tagged antibodies against B2MG, DEP1 and NOTCH3, as measured by flow cytometry. Senescent cells were analysed after 5 days of p16 or p21 expression. Numbers indicate mean fluorescent intensity (MFI) values. (**b**) Average fold increases of MFI of the same cells when senescence is induced. Experiments were performed in triplicate. Error bars show S.D.

Using the information from the validation experiments described above, we chose two of the novel membrane proteins (DEP1 and B2MG) to define a simple and specific protocol using flow cytometry that would allow for the rapid detection of senescent cells in culture. DEP1 and B2MG were selected because they had large predicted extracellular epitopes recognized by commercially available fluorescent-tagged antibodies. As a positive control, we used NOTCH3, which fulfils the same requirements and it is already known to be induced in senescent cells.^38^ Non-permeabilized cells were exposed to a mix of three fluorescently tagged antibodies and the fluorescence intensity of the sample was measured by a cytometer (see Materials and methods section for protocol details). The total time needed to measure the presence of senescent cells in cell cultures was under 2 h. As shown in Figure 5, there was a consistent two to threefold increase in the mean fluorescence intensity of all markers in EJp16 induced to senesce. We confirmed this result using another model of p21-induced senescence HT1080p21-9 (refs 41, 42) (see SA-*β*-Gal staining in Supplementary Figure 1C), which showed a ~3-fold increase of all three markers. Selective expression of these and other markers in HT1080p21-9 was also confirmed by western blot (Supplementary Figures 2C and D). Moreover, normal human diploid fibroblasts that entered replicative senescence after serial passaging also showed upregulation of the markers, although at lower levels (Figure 5), consistent with a lower percentage of SA-*β*-Gal-positive cells (see Supplementary Figure 1C). Of note, a control staining with a fluorescently tagged actin antibody did not show any increase in expression after the induction of senescence in any of these cells (Supplementary Figure 3A). These results together confirm that validated membrane markers of senescence from our proteomic screen can be successfully used to determine the presence of senescent cells in samples and could provide a faster and more selective detection tool than those currently available.

### Establishing the clinical relevance of the validated markers

**Figure 6:**
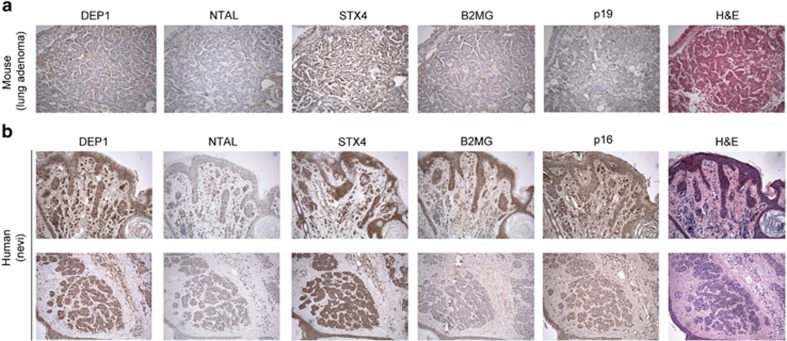
Expression of putative senescent markers in mouse and human tissues. Immunohistochemical staining of mouse (**a**) and human skin samples (**b**) with DEP1, NTAl, STX4 and B2MG antibodies. p16 is used as a known marker of senescence. Magnification: × 10 (mouse) and × 20 (human)

We next expanded our *in vitro* results to tissue obtained from mouse models and human biopsies. Figure 6a shows that lung adenomas in ^V600E^BRAF mutant mice, which have been shown to consist mostly of senescent cells,^5^ are positive for DEP1, STX4 and B2MG expression, whereas they are only weakly positive for NTAL. Of note, the level of expression of these markers was comparable to that of p16, a commonly used senescent marker. Non-adenoma cells were negative for all markers (data not shown). Moreover, human naevi, which are rich in senescent melanocytes,^9^ also showed positive staining for the same markers, especially DEP1 and STX4 (Figure 6b and Supplementary Figure 3B). STX4 also reacted with other cell types, thus showing a higher background than DEP1. This indicates that proteins in our screen can also be used to detect senescent cells in malignant and pre-malignant lesions using immunohistochemistry techniques.

**Figure 7:**
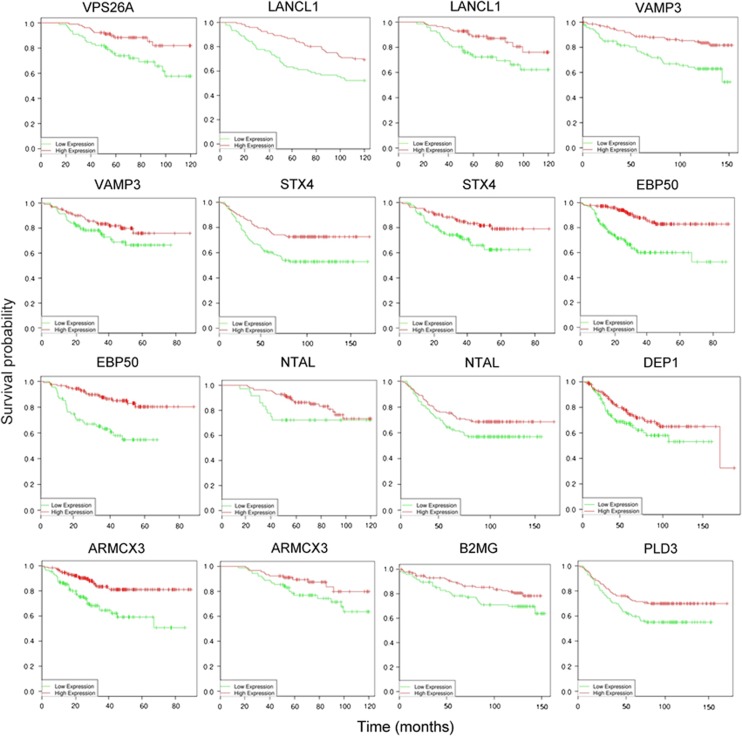
Correlation between senescent markers expression and survival in breast cancer. Kaplan–Meier survival curves of patients with breast cancer, segregated according to high (red) or low (green) expression of the genes from our panel of senescent markers, obtained from public databases through a bioinformatics analysis using PPISURV (www.bioprofiling.de). Each graph represents a different GEO data set

All these data together suggest that our panel of markers could be used clinically to detect the presence of senescent cells in tissues and thus provide diagnostic and/or prognostic information for diseases such as cancer. To confirm this hypothesis, we used PPISURV, a novel online tool that correlates gene expression with survival rates in cancer patients using publicly available data.^43^ As shown in Supplementary Table 1, high expression of our validated markers correlated with increased survival in glioma, liposarcoma, chronic lymphocytic leukaemia, colon, breast and lung cancers, among other gene expression omnibus (GEO) data sets. This is consistent with senescence being an important tumour-suppressor mechanism *in vivo.*^2^ Of note, negative correlations were also observed, suggesting that the prognostic potential of some targets may be tumour specific. Indeed, breast cancer showed the strongest correlation with the expression of our markers, as all 10 were associated with increased patient survival in different data sets (Figure 7). Interestingly, two data sets of breast cancer showed a better prognostic associated with the combined increased expression of four to six of the markers together (Supplementary Table 2). This indicates that the panel of senescent markers that we describe here could be used as a prognostic tool in cancer and underscores the clinical relevance of our findings.

## Discussion

Senescence is a well-defined cellular mechanism with a critical role in processes such as ageing^44^ and cancer.^45^ Despite having been studied for decades, the mechanisms involved in senescence are not fully understood. One of the features of senescent cells that had not been previously characterized was the profile of expression of proteins on their surface. Such proteins have the potential to be especially relevant for three reasons. First, they could contribute to define the interactions of these cells with the microenvironment and help explain how the mechanisms of senescent cell clearance work. This is important in the context of the tumour-suppressor functions of senescence, as well as its involvement in the symptoms associated with ageing.^46, 47^ Second, specific cell membrane proteins with extracellular epitopes would be useful to rapidly detect senescent cells. Given the fact that the current protocols for these analyses are not ideal, identifying extracellular epitopes of the senescent proteome could greatly improve this field of study. Finally, uncovering novel upregulated proteins could enhance our understanding of the processes that determine the senescent phenotype.

Using a proteomics approach, we identified an average of 935 proteins associated with the plasma membrane of either control or senescent EJp21 and EJp16 cells, with 107 being specific of the senescent cells. From this screen, we then selected for validation 10 proteins that were preferentially expressed in both senescent cells (and not in either of the controls) or highly expressed in one of them. Some, like the DEP1 phosphatase, has already been associated with tumour-suppressor mechanisms.^48^ Others, such as STX4, VAMP3, VPS26A and PLD3, may all have a role in vesicle trafficking in the cell,^49, 50, 51, 52, 53, 54^ perhaps contributing this way to some aspects of the SASP. We are currently performing further experiments to determine whether any of these proteins actively participates in senescence or their expression is an epiphenomenon.

We studied the expression of these targets in different models, mainly two inducible EJ cell lines that undergo senescence through activation of only one of the main pathways involved in the process, those mediated by p16 or p21. The proteins were upregulated in at least one of the models, with some clearly induced in both. Moreover, the results were also validated in normal human fibroblasts, thus confirming the relevance of the data in both replicative and stress-induced pathways of senescence. Our data suggest that these 10 proteins have the potential to be used as markers of senescence, perhaps together with those previously described (p21, p16, p15, DCR2, NOTCH3, etc.). It is likely that their expression profile would differ between tissues and depending of the triggering stimuli. For instance, EBP50 and STX4 are better induced in the p21 model, whereas DEP1, NTAL and ARMCX3 seem more specific for p16-induced senescence. Additional studies will be required to determine which combination of markers particularly defines senescent cells in each situation. This would greatly increase the specificity of any protocols to identify these cells *in vitro* and *in vivo*.

DEP1, NTAL, ARMCX3, LANCL1, B2MG, PLD3 and VPS26A have at least one predicted extracellular domain. This suggests that they could be detected with specific antibodies without the need to permeabilize cells. Using this information, we selected two of them, DEP1 and B2MG, to develop a proof of principle staining protocol that could help determine the amount of senescent cells present in a sample. The goal was to achieve higher specificity and shorter experimental times than the current standard, the SA-*β*-Gal staining assay, which has many false positives and it is not proportional to the intensity of the arrest. We believe that our results show that a fast detection method based on specific antibodies against extracellular epitopes is feasible. As mentioned above, further optimization will be needed to decide the best targets and conditions for different cell types and tissues. Increasing the simultaneous number of markers detected could also augment the specificity of the protocol, if needed. Moreover, markers specific to either the p16 or p21 pathways could help determine which of the two is preferentially activated in response to each senescence-inducing stimulus.

As senescence stops the progression of cancer *in vivo*^2^ and it is known to be increased in response to many therapies,^45^ the presence of senescent cells in tumours could be considered an indication of a controlled or less aggressive/advanced disease. Thus, we reasoned that our proteins could have a utility as prognostic tools in different types of cancer. We demonstrated this using a bioinformatics approach. We assessed a clinical application of the validated markers uncovering a positive correlation between their expression and increased survival in several malignancies. This shows that the characterization of novel markers of senescence has not only an experimental relevance in the lab but also a potential impact at the bedside. Indeed, our results suggest that the detection of senescent cells in cancer samples using one or more of our markers could be used to predict survival in breast cancer, and perhaps also in other types of tumours.

In summary, our results provide new information regarding the mechanisms involved in senescence, and we showed that this can be used experimentally to rapidly detect senescent cells, with important clinical implications. The exact role of these markers in the senescent pathways will be investigated in the future, thus contributing to our better understanding of this intricate cellular process. Such information could be important to define new therapeutic interventions that could increase the positive impact of senescence on human health and/or diminish its negative effects.

## Materials and Methods

### Cell culture

EJp21 were maintained in DMEM supplemented with 10% foetal bovin serum (FBS, Gibco, Paisley, UK), penicillin–streptomycin (50 unit/ml), hygromycin (100 *μ*g/ml) and genticin (750 *μ*g/ml). EJp16 cells were maintained in DMEM supplemented with 10% FBS, penicillin–streptomycin (50 unit/ml), hygromycin (100 *μ*g/ml) and puromycin (2 *μ*g/ml). In order to inhibit p21 or p16 expression, tet was added to the medium every 3 days to a final concentration of 1 *μ*g/ml. To induce p21 and p16 expression, cells were washed three times and seeded directly in culture medium in the absence of tet.^37^ IMR90 (human fibroblasts derived from lungs of a 16-week female foetus) and normal human diploid fibroblasts (Cellworks, San Jose, CA, USA) were maintained in DMEM supplemented with 10% FBS, and penicillin–streptomycin (50 unit/ml) until they reached replicative senescence. HT1080p21 were maintained in in DMEM supplemented with 10% FBS and penicillin–streptomycin (50 unit/ml). To induce p21 expression, 100 *μ*M isopropyl *β*-D-1-thiogalactopyranoside was added to the medium. To induce ras expression, cells were infected with a retroviral construct containing ras (gift of Stuart A Aaronson, Mount Sinai School of Medicine, New York, NY, USA).

### Plasma membrane protein extraction

This protocol was performed according to the Abcam Plasma Membrane Protein Extraction Kit (ab65400; Abcam, Cambridge, UK).

### SDS-PAGE separation, extraction and analysis of proteins from gel lanes by data-independent LC/MSE mass spectrometry

Senescent and growing EJp21 and EJp16 plasma membrane samples were separated by 10% SDS-PAGE. After staining with the Coomassie blue, the gel was cut to obtain separate sample lanes. Gel lanes were cut sequentially into slices of approximately 1.5 mm and transferred to a 96-well low binding PCR plate. Each slice was destained, digested with trypsin and peptides extracted for Mass Spectrometry analysis as previously described.^55^ Nanoscale LC was used to separate the complex peptide mixtures using a Waters nanoACQUITY UPLC (Waters, Manchester UK). Chromatography was performed using a 50 min reversed-phase gradient (formic acid (0.1%)/acetonitrile) and a 75 *μ*m × 25 cm C18 column (Waters, BE130) operated at 300 nl/min. Mass spectrometry analysis was performed using a SYNAPT G2S (Waters) operated in a data-independent (MSE) manner. The selected analysis mode enabled precursor and fragment ions from the tryptic digest to be analysed simultaneously. The data acquired were processed and searched using ProteinLynx Global Server (Waters) and visualized and reanalyzed using Scaffold (Proteome Software, Portland, OR, USA).

### SA-*β*-Gal staining

Cells were washed three times with PBS and fixed with 4% formaldehyde for 5 min at room temperature, then stained as previously described.^34^

### Immunoblot analysis

In all, 1 *μ*g/ml Protease Inhibitor Cocktail Set III (Calbiochem, Billerica, MA, USA) was added to cell lysates. Protein concentrations were determined using Bradford protein assay (Fermentas, Thermo Scientific, Waltham, MA, USA). Twenty microgram of total protein per sample was subjected to 10% or 6% SDS-PAGE and transferred to Immobilon-P membranes (Millipore, Billerica, MA, USA). An ECL detection system (Thermo Scientific) was used to visualize the results. Alternatively, an Odyssey CLx Infrared Imaging System (Li-COR, Lincoln, NE, USA) was used. See Supplementary Table 1 for antibodies used.

### Immunofluorescence

Cells were split into six-well plates containing sterile coverslips. After 24 h, media was aspirated from the plates and cells were washed three times with PBS. Cells were fixed using 1 ml of 4% paraformaldehyde for 30 min with gentle shaking. After fixing, cells were washed three times with PBS and permeabilized with 1 ml 0.1% Triton X-100 for 10 min. Cells were then washed three times with PBS and blocked with 1% BSA for 30 min. Coverslips were incubated with 100 *μ*l 1 : 100 primary antibody overnight at 4 °C. The following day, coverslips were washed three times with PBS and incubated with 100 *μ*l secondary anti-rabbit or anti-mouse antibodies (Alexa Fluor 488 and 594, Invitrogen, Paisley, UK) for 45 min in the dark. After incubation, coverslips were washed three times with PBS and stained with 4′,6-diamidino-2-phenylindole, dihydrochloride (DAPI, Invitrogen) for 10 min. Slides were labelled and the coverslips were mounted and sealed with transparent nail varnish. Slides were analysed using a Nokia TE300 semi-automatic microscope (Nokia, Keilaniemi, Finland). See Supplementary Table 1 for antibodies used.

### Immunohistochemistry

Lung adenoma (from a conditional ^V600E^BRAFknock-in mouse model)^5^ and human naevi (from clinical samples obtained by GSS) were fixed, paraffin-embedded, sectioned and stained with haematoxylin and eosin following standard protocols. Tissue immunostaining was performed as previously described.^56^ See Supplementary Table 1 for antibodies used.

### FACS analysis of senescent-associated cell surface proteins

Plates were washed with cold PBS and cells were collected by gently scraping them in 0.5 ml cold PBS, and then kept on ice. The use of trypsin was avoided to prevent internalization of extracellular proteins. Cells were centrifuged (200 *g* for 5 min at 4 °C) and the supernatant discarded. Cells were then resuspended in 200 *μ*l of blocking buffer (0.5% BSA in PBS) and incubated 15 min on ice, then transferred to 96 rounded bottom multi-well plate. These were centrifuged (500* g* for 5 min at 4 °C) and the supernatant was discarded. Cells were resuspended with a mix of the required antibodies (see Supplementary Table 1 for antibodies used), appropriately diluted, and incubated at 4 °C in the dark for 30–45 min. Cells were next washed twice with blocking buffer (150 *μ*l per well) and centrifuged for 500 *g* for 5 min at 4 °C. The cell pellet was then resuspended in 300–500 *μ*l of blocking buffer and fluorescence was read by a flow cytometer.

### Sucrose gradient and cell fractionation

Cells were washed twice at 300 *g* for 5 min with ice-cold PBS-MC (1x PBS, MgCl_2_, 1 mM Ca Cl_2_). Then, they were resuspended in 1 ml ice-cold Hypotonic Buffer (RSB: 10 mM HEPES-KOH, 10 mM KCl, 1.5 mM, MgCl_2_, pH 7.5) containing complete Protease Inhibitor Cocktail (EDTA), 1 mM activated Na_3_VO_4_, 10 mM NaF, 10 *μ*M MG132 and 5 mM *N*-ethylmaleimide and incubated for 10 min. Cells were ruptured using an ice-cold dounce homogenizer (approximately 40 strokes). To monitor cell disruption, Trypan blue and a haemocytometer were used. Samples were centrifuged at 500 × *g* for 10 min at 4 °C. 12 ml 10–50% Sucrose Density Gradients for SW40 Ti Rotor were prepared using a Biocomp Gradient Stationn automated gradient marker (Biocomp, San Antonio, TX, USA). Buffer 1: 10 mM HEPES-KOH, 1 mM MgCl_2_, 10% (W/V) sucrose, pH 7.4; buffer 2: 10 mM HEPES-KOH, 1 mM MgCl_2_, 50% (W/V) sucrose, pH 7.4. Sucrose gradients were kept on ice for 10 min before loading the homogenate carefully on top to minimize gradient disruption. The tubes were balanced and loaded into SW40 Ti buckets. Centrifugation was performed at 100 000 *g* for 18 h at 4 °C. After that, the gradients were separated into 24 × 0.5 ml fractions. Finally, 50–100 *μ*l were transferred to 96-well plates and 30 *μ*l of 4x Laemmli Sample Buffer were added before loading into gels.

## Abbreviations

FACS: fluorescence-activated cell sorting
SASP: senescence-associated secretory phenotype
SAHF: senescence-associated heterochromatic foci
SA-*β*-Gal: senescence-associated *β*-galactosidase
GEO: gene expression omnibus
tet: tetracycline
DAPI: 4′,6-diamidino-2-phenylindole, dihydrochloride

## References

[bib1] Lowe SW Cepero E Evan G Intrinsic tumour suppression Nature 2004 432 307 315 15549092 10.1038/nature03098

[bib2] Collado M Serrano M Senescence in tumours: evidence from mice and humans Nat Rev Cancer 2010 10 51 57 20029423 10.1038/nrc2772 PMC3672965

[bib3] Kuilman T Michaloglou C Mooi WJ Peeper DS The essence of senescence Genes Dev 2010 24 2463 2479 21078816 10.1101/gad.1971610 PMC2975923

[bib4] Campisi J d'Adda di Fagagna F Cellular senescence: when bad things happen to good cells Nat Rev Mol Cell Biol 2007 8 729 740 17667954 10.1038/nrm2233

[bib5] Dankort D Filenova E Collado M Serrano M Jones K McMahon M A new mouse model to explore the initiation, progression, and therapy of BRAFV600E-induced lung tumors Genes Dev 2007 21 379 384 17299132 10.1101/gad.1516407 PMC1804325

[bib6] Sarkisian CJ Keister BA Stairs DB Boxer RB Moody SE Chodosh LA Dose-dependent oncogene-induced senescence *in vivo* and its evasion during mammary tumorigenesis Nat Cell Biol 2007 9 493 505 17450133 10.1038/ncb1567

[bib7] Majumder PK Grisanzio C O'Connell F Barry M Brito JM Xu Q A prostatic intraepithelial neoplasia-dependent p27 Kip1 checkpoint induces senescence and inhibits cell proliferation and cancer progression Cancer Cell 2008 14 146 155 18691549 10.1016/j.ccr.2008.06.00 PMC2583442

[bib8] Chen Z Trotman LC Shaffer D Lin HK Dotan ZA Niki M Crucial role of p53-dependent cellular senescence in suppression of Pten-deficient tumorigenesis Nature 2005 436 725 730 16079851 10.1038/nature03918 PMC1939938

[bib9] Michaloglou C Vredeveld LC Soengas MS Denoyelle C Kuilman T van der Horst CM BRAFE600-associated senescence-like cell cycle arrest of human naevi Nature 2005 436 720 724 16079850 10.1038/nature03890

[bib10] Collado M Gil J Efeyan A Guerra C Schuhmacher AJ Barradas M Tumour biology: senescence in premalignant tumours Nature 2005 436 642 16079833 10.1038/436642a

[bib11] Herbig U Ferreira M Condel L Carey D Sedivy JM Cellular senescence in aging primates Science 2006 311 1257 16456035 10.1126/science.1122446

[bib12] Wang C Jurk D Maddick M Nelson G Martin-Ruiz C von Zglinicki T DNA damage response and cellular senescence in tissues of aging mice Aging Cell 2009 8 311 323 19627270 10.1111/j.1474-9726.2009.00481.x

[bib13] Jeyapalan JC Ferreira M Sedivy JM Herbig U Accumulation of senescent cells in mitotic tissue of aging primates Mech Ageing Dev 2007 128 36 44 17116315 10.1016/j.mad.2006.11.008 PMC3654105

[bib14] Drummond-Barbosa D Stem cells, their niches and the systemic environment: an aging network Genetics 2008 180 1787 1797 19087970 10.1534/genetics.108.098244 PMC2600921

[bib15] Krtolica A Parrinello S Lockett S Desprez PY Campisi J Senescent fibroblasts promote epithelial cell growth and tumorigenesis: a link between cancer and aging Proc Natl Acad Sci USA 2001 98 12072 12077 11593017 10.1073/pnas.211053698 PMC59769

[bib16] Hayflick L Moorehead P The serial cultivation of human diploid strains Exp Cell Res 1961 25 585 621 13905658 10.1016/0014-4827(61)90192-6

[bib17] Salama R Sadaie M Hoare M Narita M Cellular senescence and its effector programs Genes Dev 2014 28 99 114 24449267 10.1101/gad.235184.113 PMC3909793

[bib18] Serrano M Lin AW McCurrach ME Beach D Lowe SW Oncogenic ras provokes premature cell senescence associated with accumulation of p53 and p16INK4a Cell 1997 88 593 602 9054499 10.1016/s0092-8674(00)81902-9

[bib19] Jarrard DF Sarkar S Shi Y Yeager TR Magrane G Kinoshita H p16/pRb pathway alterations are required for bypassing senescence in human prostate epithelial cells Cancer Res 1999 59 2957 2964 10383161

[bib20] Stein GH Drullinger LF Soulard A Dulic V Differential roles for cyclin-dependent kinase inhibitors p21 and p16 in the mechanisms of senescence and differentiation in human fibroblasts Mol Cell Biol 1999 19 2109 2117 10022898 10.1128/mcb.19.3.2109 PMC84004

[bib21] Macip S Igarashi M Berggren P Yu J Lee SW Aaronson SA Influence of induced reactive oxygen species in p53-mediated cell fate decisions Mol Cell Biol 2003 23 8576 8585 14612402 10.1128/MCB.23.23.8576-8585.2003 PMC262651

[bib22] Macip S Igarashi M Fang L Chen A Pan ZQ Lee SW Inhibition of p21-mediated ROS accumulation can rescue p21-induced senescence EMBO J 2002 21 2180 2188 11980715 10.1093/emboj/21.9.2180 PMC125979

[bib23] Acosta JC O'Loghlen A Banito A Guijarro MV Augert A Raguz S Chemokine signaling via the CXCR2 receptor reinforces senescence Cell 2008 133 1006 1018 18555777 10.1016/j.cell.2008.03.038

[bib24] Krtolica A Parrinello S Lockett S Desprez P-Y Campisi J Senescent fibroblasts promote epithelial cell growth and tumorigenesis: a link between cancer and aging Proc Natl Acad Sci USA 2001 98 12072 12077 11593017 10.1073/pnas.211053698 PMC59769

[bib25] Mantovani A Chemokines in neoplastic progression Sem Cancer Biol 2004 14 147 148 10.1016/j.semcancer.2003.10.010 15246048

[bib26] Castro ME Ferrer I Cascon A Guijarro MV Lleonart M Ramon y Cajal S PPP1CA contributes to the senescence program induced by oncogenic Ras Carcinogenesis 2008 29 491 499 18204081 10.1093/carcin/bgm246

[bib27] Kondoh H Lleonart ME Gil J Wang J Degan P Peters G Glycolytic enzymes can modulate cellular life span Cancer Res 2005 65 177 185 15665293

[bib28] Zhang H Cohen SN Smurf2 up-regulation activates telomere-dependent senescence Genes Dev 2004 18 3028 3040 15574587 10.1101/gad.1253004 PMC535914

[bib29] Wang W Chen JX Liao R Deng Q Zhou JJ Huang S Sequential activation of the MEK-extracellular signal-regulated kinase and MKK3/6-p38 mitogen-activated protein kinase pathways mediates oncogenic ras-induced premature senescence Mol Cell Biol 2002 22 3389 3403 11971971 10.1128/MCB.22.10.3389-3403.2002 PMC133789

[bib30] van Deursen JM The role of senescent cells in ageing Nature 2014 509 439 446 24848057 10.1038/nature13193 PMC4214092

[bib31] Narita M Narita M Krizhanovsky V Nunez S Chicas A Hearn SA A novel role for high-mobility group a proteins in cellular senescence and heterochromatin formation Cell 2006 126 503 514 16901784 10.1016/j.cell.2006.05.052

[bib32] Narita M Nunez S Heard E Narita M Lin AW Hearn SA Rb-mediated heterochromatin formation and silencing of E2F target genes during cellular senescence Cell 2003 113 703 716 12809602 10.1016/s0092-8674(03)00401-x

[bib33] Kosar M Bartkova J Hubackova S Hodny Z Lukas J Bartek J Senescence-associated heterochromatin foci are dispensable for cellular senescence, occur in a cell type- and insult-dependent manner and follow expression of p16(ink4a) Cell Cycle 2011 10 457 468 21248468 10.4161/cc.10.3.14707

[bib34] Dimri GP Lee XH Basile G Acosta M Scott C Roskelley C A biomarker that identifies senescent human-cells in culture and in aging skin in-vivo Proc Natl Acad Sci USA 1995 92 9363 9367 7568133 10.1073/pnas.92.20.9363 PMC40985

[bib35] Lee BY Han JA Im JS Morrone A Johung K Goodwin EC Senescence-associated beta-galactosidase is lysosomal beta-galactosidase Aging Cell 2006 5 187 195 16626397 10.1111/j.1474-9726.2006.00199.x

[bib36] Yang NC Hu ML The limitations and validities of senescence associated-beta-galactosidase activity as an aging marker for human foreskin fibroblast Hs68 cells Exp Gerontol 2005 40 813 819 16154306 10.1016/j.exger.2005.07.011

[bib37] Fang L Igarashi M Leung J Sugrue MM Lee SW Aaronson SA p21Waf1/Cip1/Sdi1 induces permanent growth arrest with markers of replicative senescence in human tumor cells lacking functional p53 Oncogene 1999 18 2789 2797 10362249 10.1038/sj.onc.1202615

[bib38] Gorgoulis VG Pratsinis H Zacharatos P Demoliou C Sigala F Asimacopoulos PJ p53-dependent ICAM-1 overexpression in senescent human cells identified in atherosclerotic lesions Lab Invest 2005 85 502 511 15711569 10.1038/labinvest.3700241

[bib39] Cui H Kong Y Xu M Zhang H Notch3 functions as a tumor suppressor by controlling cellular senescence Cancer Res 2013 73 3451 3459 23610446 10.1158/0008-5472.CAN-12-3902 PMC3674178

[bib40] Zhang Z Rosen DG Yao JL Huang J Liu J Expression of p14ARF, p15INK4b, p16INK4a, and DCR2 increases during prostate cancer progression Mod Pathol 2006 19 1339 1343 16799475 10.1038/modpathol.3800655

[bib41] Chang BD Broude EV Fang J Kalinichenko TV Abdryashitov R Poole JC p21Waf1/Cip1/Sdi1-induced growth arrest is associated with depletion of mitosis-control proteins and leads to abnormal mitosis and endoreduplication in recovering cells Oncogene 2000 19 2165 2170 10815808 10.1038/sj.onc.1203573

[bib42] Masgras I Carrera S de Verdier PJ Brennan P Majid A Makhtar W Reactive oxygen species and mitochondrial sensitivity to oxidative stress determine induction of cancer cell death by p21 J Biol Chem 2012 287 9845 9854 22311974 10.1074/jbc.M111.250357 PMC3322987

[bib43] Antonov AV Krestyaninova M Knight RA Rodchenkov I Melino G Barlev NA PPISURV: a novel bioinformatics tool for uncovering the hidden role of specific genes in cancer survival outcome Oncogene 2014 33 1621 1628 23686313 10.1038/onc.2013.119

[bib44] Lopez-Otin C Blasco MA Partridge L Serrano M Kroemer G The hallmarks of aging Cell 2013 153 1194 1217 23746838 10.1016/j.cell.2013.05.039 PMC3836174

[bib45] Perez-Mancera PA Young AR Narita M Inside and out: the activities of senescence in cancer Nat Rev Cancer 2014 14 547 558 25030953 10.1038/nrc3773

[bib46] Baker DJ Wijshake T Tchkonia T LeBrasseur NK Childs BG van de Sluis B Clearance of p16Ink4a-positive senescent cells delays ageing-associated disorders Nature 2011 479 232 236 22048312 10.1038/nature10600 PMC3468323

[bib47] Kang TW Yevsa T Woller N Hoenicke L Wuestefeld T Dauch D Senescence surveillance of pre-malignant hepatocytes limits liver cancer development Nature 2011 479 547 551 22080947 10.1038/nature10599

[bib48] Sacco F Tinti M Palma A Ferrari E Nardozza AP Huijsduijnen RH Tumor suppressor density-enhanced phosphatase-1 (DEP-1) inhibits the RAS pathway by direct dephosphorylation of ERK1/2 kinase J Biol Chem 2009 284 22048 22058 19494114 10.1074/jbc.M109.002758 PMC2755929

[bib49] Kean MJ Williams KC Skalski M Myers D Burtnik A Foster D VAMP3, syntaxin-13 and SNAP23 are involved in secretion of matrix metalloproteinases, degradation of the extracellular matrix and cell invasion J Cell Sci 2009 122 4089 4098 19910495 10.1242/jcs.052761

[bib50] Chen YA Scheller RH SNARE-mediated membrane fusion Nat Rev Mol Cell Biol 2001 2 98 106 11252968 10.1038/35052017

[bib51] Polgar J Chung SH Reed GL Vesicle-associated membrane protein 3 (VAMP-3) and VAMP-8 are present in human platelets and are required for granule secretion Blood 2002 100 1081 1083 12130530 10.1182/blood.v100.3.1081

[bib52] Olson AL Knight JB Pessin JE Syntaxin 4, VAMP2, and/or VAMP3/cellubrevin are functional target membrane and vesicle SNAP receptors for insulin-stimulated GLUT4 translocation in adipocytes Mol Cell Biol 1997 17 2425 2435 9111311 10.1128/mcb.17.5.2425 PMC232091

[bib53] Bugarcic A Zhe Y Kerr MC Griffin J Collins BM Teasdale RD Vps26A and Vps26B subunits define distinct retromer complexes Traffic 2011 12 1759 1773 21920005 10.1111/j.1600-0854.2011.01284.x

[bib54] Osisami M Ali W Frohman MA A role for phospholipase D3 in myotube formation PLoS One 2012 7 e33341 22428023 10.1371/journal.pone.0033341 PMC3299777

[bib55] Boyd RS Jukes-Jones R Walewska R Brown D Dyer MJ Cain K Protein profiling of plasma membranes defines aberrant signaling pathways in mantle cell lymphoma Mol Cell Proteomics 2009 8 1501 1515 19346216 10.1074/mcp.M800515-MCP200 PMC2709182

[bib56] Mercer K Giblett S Green S Lloyd D DaRocha Dias S Plumb M Expression of endogenous oncogenic V600EB-raf induces proliferation and developmental defects in mice and transformation of primary fibroblasts Cancer Res 2005 65 11493 11500 16357158 10.1158/0008-5472.CAN-05-2211 PMC2640458

