# Supplementary figures and images for: Characterization of novel markers of senescence and their prognostic potential in cancer
Source: Cell Death Dis. 2014 Nov 20;5(11):e1528–. doi: 10.1038/cddis.2014.489 (PMC4260747; doi:10.1038/cddis.2014.489)

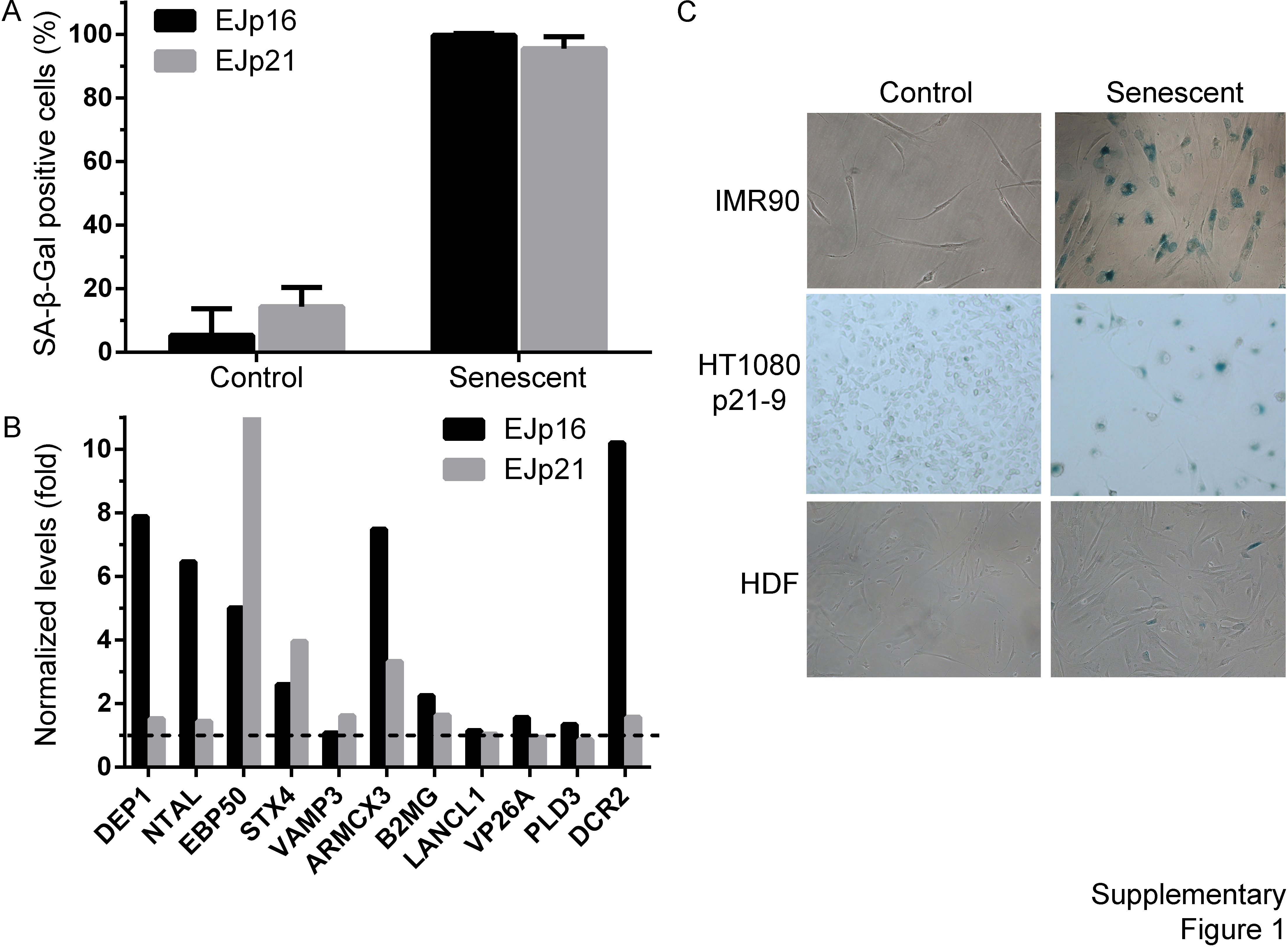

Supplement: Supplementary Figure 1 [file cddis2014489x1.tif]

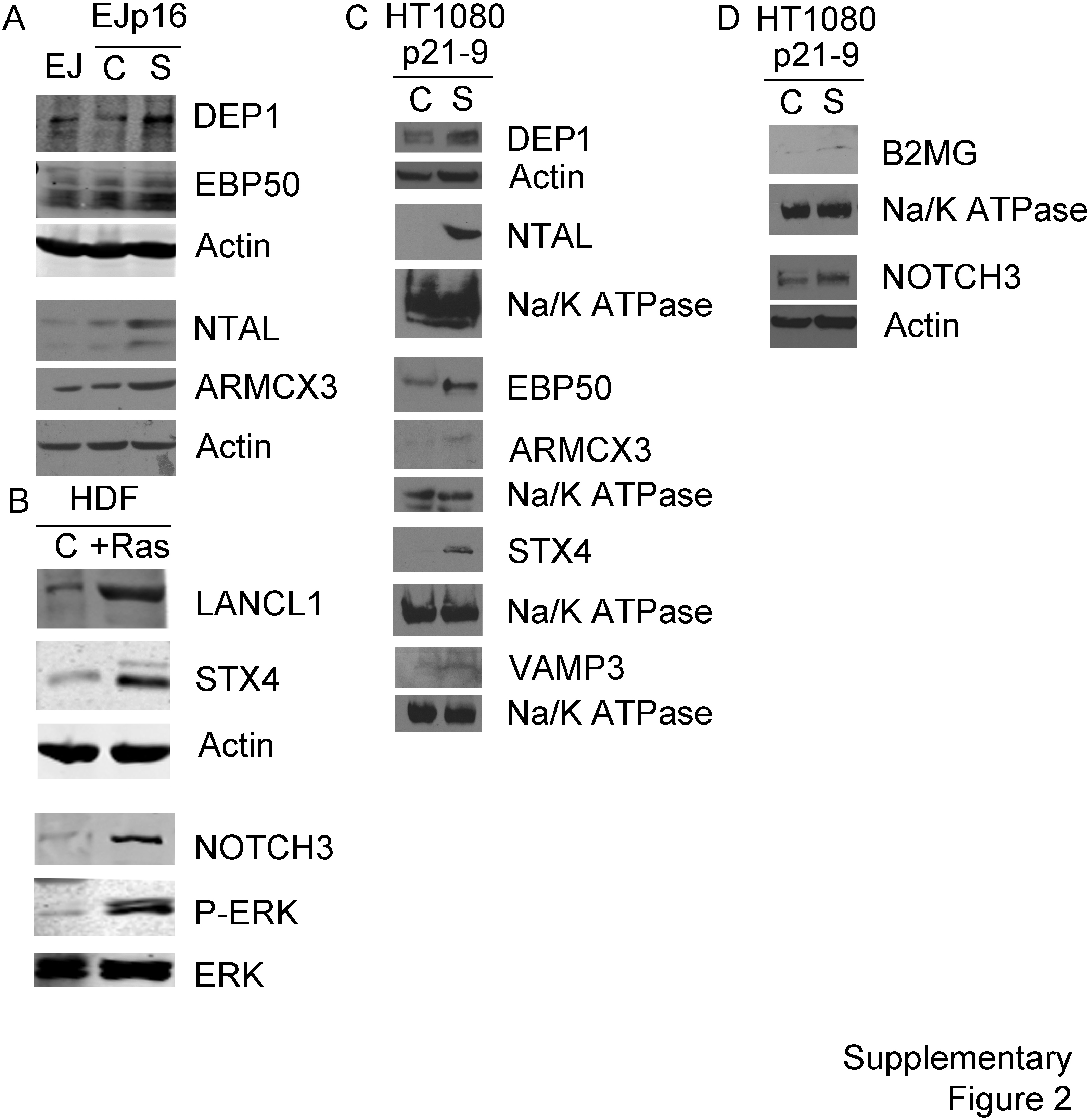

Supplement: Supplementary Figure 2 [file cddis2014489x2.tif]

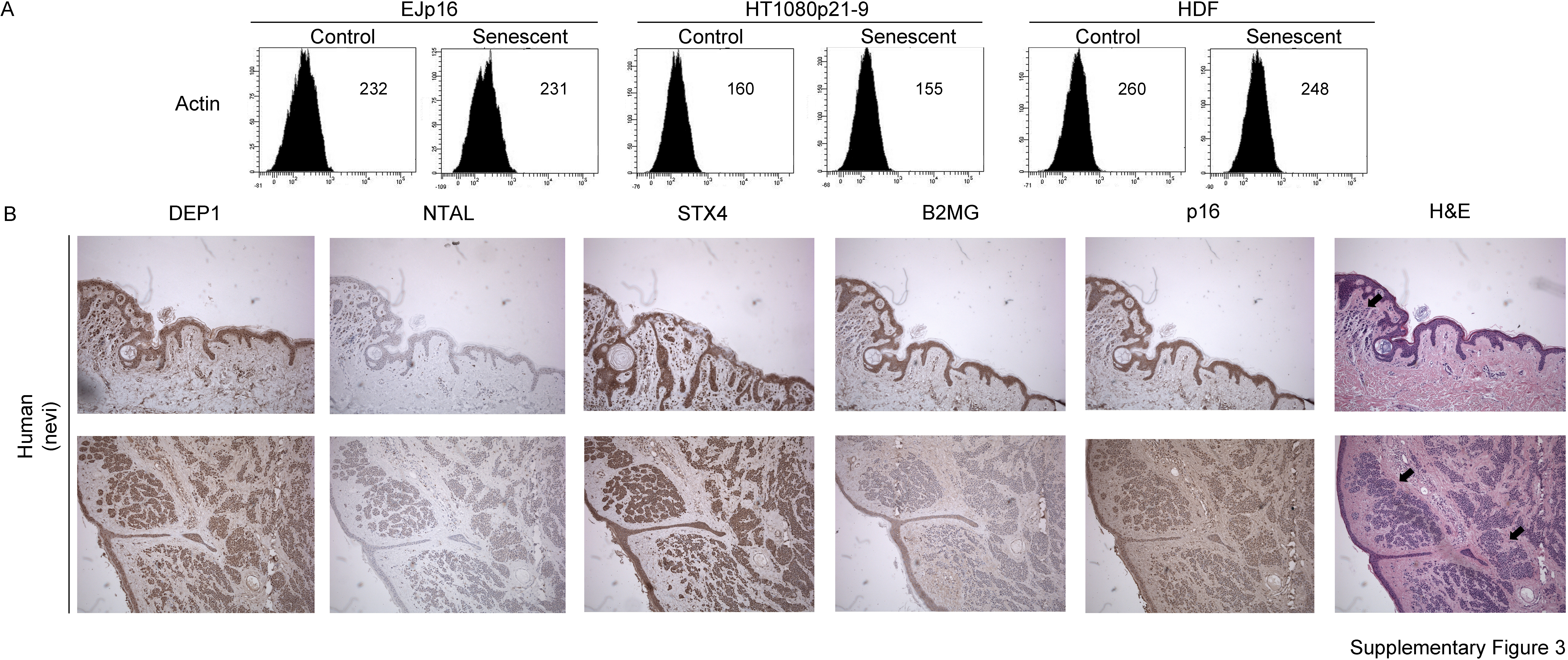

Supplement: Supplementary Figure 3 [file cddis2014489x3.tif]
