# Supplementary material for: Characterization of novel markers of senescence and their prognostic potential in cancer
Source: Cell Death Dis. 2014 Nov 20;5(11):e1528–. doi: 10.1038/cddis.2014.489 (PMC4260747; doi:10.1038/cddis.2014.489)
Supplement: Supplementary Information [file cddis2014489x4.doc]

**Supplementary Figure 1. A)** SA-β-Gal staining of EJp16 and EJp21 uninduced (Control) or 4 days after tet removal to induce the expression of exogenous p16 or p21 (Senescent). Percentages of positive cells (average of 3 independent experiments) are plotted. Error bars show standard deviation. **B)** Quantitation of the Western Blots in Figure 2. Densitometry was performed using Image J software. Intensity values were normalized to the equivalent controls (calnexin or Na/K ATPase). Fold increases between control and senescent cells are plotted. **C)** SA-β-Gal staining of control and senescent IMR90, HT1080p21-9 and normal human diploid fibroblasts (HDF). Fibroblasts (IMR90 and HDF) were induced to senesce after serial passaging, while HT1080p21-9 senesced after 4 days of p21 expression following exposure to IPTG.

**Supplementary Figure 2. Western blot validation of senescent-specific targets**. **A)** Protein expression of selected targets in whole cell lysates from parental EJ cells and EJp16, uninduced (C) or 4 days after tet removal (S). **B)** Protein expression of selected targets in whole cell lysates from human diploid fibroblasts 4 days after being infected with a Ras-expressing retrovirus. Phospho-ERK is provided as a control of activation of the Ras-MAPK pathway. **C)** and **D)** Protein expression of selected targets in lysates from HT1080p21-9 uninduced (C) or 4 days after exposure to IPTG to induce p21 expression (S). Actin and Na/K ATPase are used as loading controls.

**Supplementary Figure 3. A)** Representative plot analysis of fluorescence levels in control and senescent EJp16, HT1080p21-9 and human diploid fibroblasts (HDF) stained with a fluorescently tagged antibody against actin, as measured by flow cytometry. Senescent cells were analysed after 5 days of p16 or p21 expression. Numbers indicate Mean Fluorescent Intensity values. The experiment was performed in triplicate, with similar results (no significant changes of expression after senescence). **B)** Expression of putative senescent markers in mouse and human tissues. Alternative images of the immunohistochemical staining of human skin samples from Figure 5. Black arrows mark nevi cells. Magnification: 10X.

**Supplementary Table 1. Correlation between expression of senescent markers and survival in cancer.** The tables show the effect of expression of one of our markers **(A-I)** on the survival of different cancer types, using available GEO datasets. A Positive correlation means increased survival when a gene is highly expressed. A Negative correlation implies the opposite. Data analysed with PPISURV.

**Supplementary Table 2. Correlation between senescent markers expression and survival in cancer.** Same as supplementary Table 1 but showing a list of positively-correlated markers in two breast cancer datasets **(A** and **B)**.

**Supplementary Table 3. Antibodies used in the study.** List of antibodies used for Western Blots **(A)**, immunofluorescence **(B)**, immunohistochemistry **(C)** and flow cytometry **(D)**.

**Supplementary Table 1.**

A) VPS26A

| **GEO dataset** | **Cancer Type** | **GENE (Probe ID)** | **P-value** | **Effect Sign** |
| --- | --- | --- | --- | --- |
| [molecular subclasses of high-grade glioma: prognosis, disease progression, and neurogenesis](http://www.ncbi.nlm.nih.gov/geo/query/acc.cgi?acc=GSE4271) | high-grade glioma | 201807_AT | 0.000316 | **Positive** |
| [183 breast tumors from the helsinki univerisity central hospital with survival information](http://www.ncbi.nlm.nih.gov/geo/query/acc.cgi?acc=GSE24450) | breast cancer | ILMN_1781039 | 0.00417 | **Positive** |
| [experimentally derived metastasis gene expression profile predicts recurrence and death in colon cancer patients](http://www.ncbi.nlm.nih.gov/geo/query/acc.cgi?acc=GSE17538) | colon cancer | 243316_X_AT | 0.0157 | **Negative** |
| [whole-transcript expression data for liposarcoma](http://www.ncbi.nlm.nih.gov/geo/query/acc.cgi?acc=GSE30929) | liposarcoma | 201807_AT | 0.0162 | **Positive** |
| [expression profile-defined classification of lung adenocarcinoma](http://www.ncbi.nlm.nih.gov/geo/query/acc.cgi?acc=GSE11969) | lung cancer | 11846 | 0.0242 | **Negative** |
| [prediction of survival in diffuse large b cell lymphoma treated with chemotherapy plus rituximab](http://www.ncbi.nlm.nih.gov/geo/query/acc.cgi?acc=GSE10846) | diffuse large b cell lymphoma | 201807_AT | 0.0441 | **Positive** |
| [metastasis gene expression profile predicts recurrence and death in colon cancer patients (moffitt samples)](http://www.ncbi.nlm.nih.gov/geo/query/acc.cgi?acc=GSE17536) | colon cancer | 243316_X_AT | 0.0443 | **Negative** |
| [an eight-gene expression signature for the prediction of survival and time to treatment in chronic lymphocytic leukemia](http://www.ncbi.nlm.nih.gov/geo/query/acc.cgi?acc=GSE22762) | chronic lymphocytic leukemia | 201807_AT | 0.0611 | **Positive** |
| [analysis of early primary breast cancer to identify prognostic markers and associated pathways: mrna and mirna profiling](http://www.ncbi.nlm.nih.gov/geo/query/acc.cgi?acc=GSE22220) | breast cancer | 3800100 | 0.101 | **Positive** |

B) LANCL1

| **GEO dataset** | **Cancer Type** | **GENE (Probe ID)** | **P-value** | **Effect Sign** |
| --- | --- | --- | --- | --- |
| [prediction of survival in diffuse large b cell lymphoma treated with chemotherapy plus rituximab](http://www.ncbi.nlm.nih.gov/geo/query/acc.cgi?acc=GSE10846) | diffuse large b cell lymphoma | 202020_S_AT | 2.31e-07 | **Positive** |
| [analysis of early primary breast cancer to identify prognostic markers and associated pathways: mrna and mirna profiling](http://www.ncbi.nlm.nih.gov/geo/query/acc.cgi?acc=GSE22220) | breast cancer | 940692 | 0.000841 | **Positive** |
| [gene expression data for pathological stage i-ii lung adenocarcinomas](http://www.ncbi.nlm.nih.gov/geo/query/acc.cgi?acc=GSE31210) | lung cancer | 202019_S_AT | 0.00234 | **Negative** |
| [relapse-related molecular signature in lung adenocarcinomas identifies patients with dismal prognosis](http://www.ncbi.nlm.nih.gov/geo/query/acc.cgi?acc=GSE13213) | lung cancer | A_24_P259922 | 0.00805 | **Negative** |
| [183 breast tumors from the helsinki univerisity central hospital with survival information](http://www.ncbi.nlm.nih.gov/geo/query/acc.cgi?acc=GSE24450) | breast cancer | ILMN_2081988 | 0.00814 | **Positive** |
| [gene expression in fixed tissues and outcome in hepatocellular carcinoma](http://www.ncbi.nlm.nih.gov/geo/query/acc.cgi?acc=GSE10143) | hepatocellular carcinoma | DAP2_1985 | 0.0172 | **Negative** |
| [combining clinical, pathology, and gene expression data to predict recurrence of hepatocellular carcinoma](http://www.ncbi.nlm.nih.gov/geo/query/acc.cgi?acc=GSE20140) | hepatocellular carcinoma | DAP2_1985 | 0.0255 | **Negative** |
| [heterogeneity of response to chemotherapy and recurrence-free survival in neoadjuvant breast cancer: results from the i-spy 1 trial](http://www.ncbi.nlm.nih.gov/geo/query/acc.cgi?acc=GSE22226) | breast cancer | 41686 | 0.0262 | **Positive** |
| [endocrine sensitivity index validation dataset](http://www.ncbi.nlm.nih.gov/geo/query/acc.cgi?acc=GSE17705) | breast cancer | 202020_S_AT | 0.0454 | **Positive** |
| [molecular characterization of breast cancer subtypes derived from joint analysis of high throughput mirna and mrna data](http://www.ncbi.nlm.nih.gov/geo/query/acc.cgi?acc=GSE19783) | breast cancer | A_23_P50887 | 0.0531 | **Positive** |
| [breast cancer relapse free survival](http://www.ncbi.nlm.nih.gov/geo/query/acc.cgi?acc=GSE2034) | breast cancer | 202019_S_AT | 0.0538 | **Positive** |
| [validation cohort for genomic predictor of response and survival following neoadjuvant taxane-anthracycline chemotherapy in breast cancer](http://www.ncbi.nlm.nih.gov/geo/query/acc.cgi?acc=GSE25065) | breast cancer | 202020_S_AT | 0.0632 | **Positive** |

C) VAMP3

| **GEO dataset** | **Cancer Type** | **GENE (Probe ID)** | **P-value** | **Effect Sign** |
| --- | --- | --- | --- | --- |
| [expression data from untreated cll patients](http://www.ncbi.nlm.nih.gov/geo/query/acc.cgi?acc=GSE39671) | chronic lymphocytic leukemia | 201336_AT | 9.22e-05 | **Positive** |
| [an expression signature for p53 in breast cancer predicts mutation status, transcriptional effects, and patient survival](http://www.ncbi.nlm.nih.gov/geo/query/acc.cgi?acc=GSE3494) | breast cancer | 201336_AT | 0.000327 | **Positive** |
| [an eight-gene expression signature for the prediction of survival and time to treatment in chronic lymphocytic leukemia](http://www.ncbi.nlm.nih.gov/geo/query/acc.cgi?acc=GSE22762) | chronic lymphocytic leukemia | 201336_AT | 0.00201 | **Positive** |
| [whole-transcript expression data for liposarcoma](http://www.ncbi.nlm.nih.gov/geo/query/acc.cgi?acc=GSE30929) | liposarcoma | 211749_S_AT | 0.00303 | **Positive** |
| [the humoral immune system has a key prognostic impact in node-negative breast cancer](http://www.ncbi.nlm.nih.gov/geo/query/acc.cgi?acc=GSE11121) | breast cancer | 211749_S_AT | 0.00565 | **Positive** |
| [expression data for early stage nsclc](http://www.ncbi.nlm.nih.gov/geo/query/acc.cgi?acc=GSE19188) | lung cancer | 201337_S_AT | 0.0385 | **Positive** |
| [prediction of progression-free survival in patients with advanced-stage serous ovarian cancer](http://www.ncbi.nlm.nih.gov/geo/query/acc.cgi?acc=GSE17260) | ovarian cancer | A_24_P370887 | 0.0485 | **Negative** |
| [prediction of survival in diffuse large b cell lymphoma treated with chemotherapy plus rituximab](http://www.ncbi.nlm.nih.gov/geo/query/acc.cgi?acc=GSE10846) | diffuse large b cell lymphoma | 201336_AT | 0.0566 | **Positive** |
| [endocrine sensitivity index validation dataset](http://www.ncbi.nlm.nih.gov/geo/query/acc.cgi?acc=GSE17705) | breast cancer | 201336_AT | 0.0657 | **Positive** |
| [a prognostic gene expression index in ovarian cancer](http://www.ncbi.nlm.nih.gov/geo/query/acc.cgi?acc=GSE14764) | ovarian cancer | 201337_S_AT | 0.0856 | **Negative** |
| [search for a gene-expression signature of breast cancer local recurrence in young women](http://www.ncbi.nlm.nih.gov/geo/query/acc.cgi?acc=GSE30682) | breast cancer | ILMN_1714527 | 0.0909 | **Positive** |
| [analysis of early primary breast cancer to identify prognostic markers and associated pathways: mrna and mirna profiling](http://www.ncbi.nlm.nih.gov/geo/query/acc.cgi?acc=GSE22220) | breast cancer | 6860575 | 0.0976 | **Positive** |
| [validation cohort for genomic predictor of response and survival following neoadjuvant taxane-anthracycline chemotherapy in breast cancer](http://www.ncbi.nlm.nih.gov/geo/query/acc.cgi?acc=GSE25065) | breast cancer | 201337_S_AT | 0.0999 | **Positive** |
| [gene expression data for pathological stage i-ii lung adenocarcinomas](http://www.ncbi.nlm.nih.gov/geo/query/acc.cgi?acc=GSE31210) | lung cancer | 201336_AT | 0.102 | **Positive** |

D) STX4

| **GEO dataset** | **Cancer Type** | **GENE (Probe ID)** | **P-value** | **Effect Sign** |
| --- | --- | --- | --- | --- |
| [breast cancer relapse free survival](http://www.ncbi.nlm.nih.gov/geo/query/acc.cgi?acc=GSE2034) | breast cancer | 203530_S_AT | 0.0018 | **Positive** |
| [validation cohort for genomic predictor of response and survival following neoadjuvant taxane-anthracycline chemotherapy in breast cancer](http://www.ncbi.nlm.nih.gov/geo/query/acc.cgi?acc=GSE25065) | breast cancer | 203530_S_AT | 0.0135 | **Positive** |
| [endocrine sensitivity index validation dataset](http://www.ncbi.nlm.nih.gov/geo/query/acc.cgi?acc=GSE17705) | breast cancer | 203530_S_AT | 0.0146 | **Positive** |
| [whole-transcript expression data for liposarcoma](http://www.ncbi.nlm.nih.gov/geo/query/acc.cgi?acc=GSE30929) | liposarcoma | 203530_S_AT | 0.0195 | **Negative** |
| [maqc-ii project: multiple myeloma (mm) data set](http://www.ncbi.nlm.nih.gov/geo/query/acc.cgi?acc=GSE24080) | multiple myeloma | 229395_AT | 0.0302 | **Negative** |
| [a gene expression signature identifies two prognostic subgroups of basal breast cancer](http://www.ncbi.nlm.nih.gov/geo/query/acc.cgi?acc=GSE21653) | breast cancer | 236232_AT | 0.0521 | **Positive** |
| [human lung adenocarcinoma](http://www.ncbi.nlm.nih.gov/geo/query/acc.cgi?acc=GSE36471) | lung cancer | 16629 | 0.0527 | **Positive** |
| [prediction of survival in diffuse large b cell lymphoma treated with chemotherapy plus rituximab](http://www.ncbi.nlm.nih.gov/geo/query/acc.cgi?acc=GSE10846) | diffuse large b cell lymphoma | 236232_AT | 0.0533 | **Negative** |
| [genome-wide profiling of astrocytic gliomas](http://www.ncbi.nlm.nih.gov/geo/query/acc.cgi?acc=GSE18166) | astrocytic gliomas | 29067 | 0.0576 | **Negative** |
| [down-regulation of ecrg4, a candidate tumor suppressor gene in human breast cancer](http://www.ncbi.nlm.nih.gov/geo/query/acc.cgi?acc=GSE31448) | breast cancer | 236232_AT | 0.125 | **Positive** |

E) EBP50

| **GEO dataset** | **Cancer Type** | **GENE (Probe ID)** | **P-value** | **Effect Sign** |
| --- | --- | --- | --- | --- |
| [discovery cohort for genomic predictor of response and survival following neoadjuvant taxane-anthracycline chemotherapy in breast cancer](http://www.ncbi.nlm.nih.gov/geo/query/acc.cgi?acc=GSE25055) | breast cancer | 201349_AT | 6.56e-08 | **Positive** |
| [validation cohort for genomic predictor of response and survival following neoadjuvant taxane-anthracycline chemotherapy in breast cancer](http://www.ncbi.nlm.nih.gov/geo/query/acc.cgi?acc=GSE25065) | breast cancer | 201349_AT | 1.5e-05 | **Positive** |
| [search for a gene-expression signature of breast cancer local recurrence in young women](http://www.ncbi.nlm.nih.gov/geo/query/acc.cgi?acc=GSE30682) | breast cancer | ILMN_1680925 | 0.000439 | **Positive** |
| [a gene signature predicting for survival in suboptimally debulked patients with ovarian cancer](http://www.ncbi.nlm.nih.gov/geo/query/acc.cgi?acc=GSE26712) | ovarian cancer | 201349_AT | 0.000987 | **Positive** |
| [heterogeneity of response to chemotherapy and recurrence-free survival in neoadjuvant breast cancer: results from the i-spy 1 trial](http://www.ncbi.nlm.nih.gov/geo/query/acc.cgi?acc=GSE22226) | breast cancer | 30440 | 0.00485 | **Positive** |
| [combining clinical, pathology, and gene expression data to predict recurrence of hepatocellular carcinoma](http://www.ncbi.nlm.nih.gov/geo/query/acc.cgi?acc=GSE20140) | hepatocellular carcinoma | DAP3_6052 | 0.0413 | **Negative** |
| [an eight-gene expression signature for the prediction of survival and time to treatment in chronic lymphocytic leukemia](http://www.ncbi.nlm.nih.gov/geo/query/acc.cgi?acc=GSE22762) | chronic lymphocytic leukemia | 201349_AT | 0.0454 | **Positive** |
| [gene expression profiling in breast cancer: understanding the molecular basis of histologic grade to improve prognosis](http://www.ncbi.nlm.nih.gov/geo/query/acc.cgi?acc=GSE2990) | breast cancer | 201349_AT | 0.0607 | **Negative** |
| [maqc-ii project: multiple myeloma (mm) data set](http://www.ncbi.nlm.nih.gov/geo/query/acc.cgi?acc=GSE24080) | multiple myeloma | 201349_AT | 0.0964 | **Negative** |
| [prediction of progression-free survival in patients with advanced-stage serous ovarian cancer](http://www.ncbi.nlm.nih.gov/geo/query/acc.cgi?acc=GSE17260) | ovarian cancer | A_23_P308519 | 0.0971 | **Positive** |
| [gene expression in fixed tissues and outcome in hepatocellular carcinoma](http://www.ncbi.nlm.nih.gov/geo/query/acc.cgi?acc=GSE10143) | hepatocellular carcinoma | DAP3_6052 | 0.0998 | **Negative** |
| [breast cancer relapse free survival](http://www.ncbi.nlm.nih.gov/geo/query/acc.cgi?acc=GSE2034) | breast cancer | 201349_AT | 0.114 | **Positive** |

F) NTAL

| **GEO dataset** | **Cancer Type** | **GENE (Probe ID)** | **P-value** | **Effect Sign** |
| --- | --- | --- | --- | --- |
| [the humoral immune system has a key prognostic impact in node-negative breast cancer](http://www.ncbi.nlm.nih.gov/geo/query/acc.cgi?acc=GSE11121) | breast cancer | 211768_AT | 0.000251 | **Negative** |
| [genome-wide profiling of astrocytic gliomas](http://www.ncbi.nlm.nih.gov/geo/query/acc.cgi?acc=GSE18166) | astrocytic gliomas | 25534 | 0.00151 | **Positive** |
| [metastasis gene expression profile predicts recurrence and death in colon cancer patients (moffitt samples)](http://www.ncbi.nlm.nih.gov/geo/query/acc.cgi?acc=GSE17536) | colon cancer | 211768_AT | 0.00361 | **Positive** |
| [prediction of progression-free survival in patients with advanced-stage serous ovarian cancer](http://www.ncbi.nlm.nih.gov/geo/query/acc.cgi?acc=GSE17260) | ovarian cancer | A_24_P940327 | 0.0148 | **Positive** |
| [molecular subclasses of high-grade glioma: prognosis, disease progression, and neurogenesis](http://www.ncbi.nlm.nih.gov/geo/query/acc.cgi?acc=GSE4271) | high-grade glioma | 211768_AT | 0.0267 | **Negative** |
| [human lung adenocarcinoma](http://www.ncbi.nlm.nih.gov/geo/query/acc.cgi?acc=GSE36471) | lung cancer | 25299 | 0.0318 | **Positive** |
| [relapse-related molecular signature in lung adenocarcinomas identifies patients with dismal prognosis](http://www.ncbi.nlm.nih.gov/geo/query/acc.cgi?acc=GSE13213) | lung cancer | A_24_P940327 | 0.0491 | **Positive** |
| [183 breast tumors from the helsinki univerisity central hospital with survival information](http://www.ncbi.nlm.nih.gov/geo/query/acc.cgi?acc=GSE24450) | breast cancer | ILMN_1803560 | 0.0576 | **Positive** |
| [gene expression signatures for predicting prognosis of squamous cell lung carcinomas](http://www.ncbi.nlm.nih.gov/geo/query/acc.cgi?acc=GSE4573) | lung cancer | 211768_AT | 0.0591 | **Negative** |
| [breast cancer relapse free survival](http://www.ncbi.nlm.nih.gov/geo/query/acc.cgi?acc=GSE2034) | breast cancer | 221581_S_AT | 0.0723 | **Positive** |
| [strong time dependence of the 76-gene prognostic signature](http://www.ncbi.nlm.nih.gov/geo/query/acc.cgi?acc=GSE7390) | breast cancer | 221581_S_AT | 0.0724 | **Positive** |
| [experimentally derived metastasis gene expression profile predicts recurrence and death in colon cancer patients](http://www.ncbi.nlm.nih.gov/geo/query/acc.cgi?acc=GSE17538) | colon cancer | 211768_AT | 0.115 | **Positive** |

G) DEP1

| **GEO dataset** | **Cancer Type** | **GENE (Probe ID)** | **P-value** | **Effect Sign** |
| --- | --- | --- | --- | --- |
| [strong time dependence of the 76-gene prognostic signature](http://www.ncbi.nlm.nih.gov/geo/query/acc.cgi?acc=GSE7390) | breast cancer | 214137_AT | 0.00417 | **Negative** |
| [prediction of survival in diffuse large b cell lymphoma treated with chemotherapy plus rituximab](http://www.ncbi.nlm.nih.gov/geo/query/acc.cgi?acc=GSE10846) | diffuse large b cell lymphoma | 214137_AT | 0.0056 | **Negative** |
| [human lung adenocarcinoma](http://www.ncbi.nlm.nih.gov/geo/query/acc.cgi?acc=GSE36471) | lung cancer | 31430 | 0.0088 | **Negative** |
| [an eight-gene expression signature for the prediction of survival and time to treatment in chronic lymphocytic leukemia](http://www.ncbi.nlm.nih.gov/geo/query/acc.cgi?acc=GSE22762) | chronic lymphocytic leukemia | 210173_AT | 0.0104 | **Negative** |
| [gene expression signatures for predicting prognosis of squamous cell lung carcinomas](http://www.ncbi.nlm.nih.gov/geo/query/acc.cgi?acc=GSE4573) | lung cancer | 210173_AT | 0.0139 | **Positive** |
| [expression data from untreated cll patients](http://www.ncbi.nlm.nih.gov/geo/query/acc.cgi?acc=GSE39671) | chronic lymphocytic leukemia | 227396_AT | 0.0279 | **Negative** |
| [validation cohort for genomic predictor of response and survival following neoadjuvant taxane-anthracycline chemotherapy in breast cancer](http://www.ncbi.nlm.nih.gov/geo/query/acc.cgi?acc=GSE25065) | breast cancer | 214137_AT | 0.0461 | **Negative** |
| [a gene expression signature identifies two prognostic subgroups of basal breast cancer](http://www.ncbi.nlm.nih.gov/geo/query/acc.cgi?acc=GSE21653) | breast cancer | 227396_AT | 0.0733 | **Positive** |
| [endocrine sensitivity index validation dataset](http://www.ncbi.nlm.nih.gov/geo/query/acc.cgi?acc=GSE17705) | breast cancer | 214137_AT | 0.0902 | **Negative** |
| [down-regulation of ecrg4, a candidate tumor suppressor gene in human breast cancer](http://www.ncbi.nlm.nih.gov/geo/query/acc.cgi?acc=GSE31448) | breast cancer | 227396_AT | 0.102 | **Positive** |

H) ARMCX3

| **GEO dataset** | **Cancer Type** | **GENE (Probe ID)** | **P-value** | **Effect Sign** |
| --- | --- | --- | --- | --- |
| [discovery cohort for genomic predictor of response and survival following neoadjuvant taxane-anthracycline chemotherapy in breast cancer](http://www.ncbi.nlm.nih.gov/geo/query/acc.cgi?acc=GSE25055) | breast cancer | 217858_S_AT | 5.92e-05 | **Positive** |
| [prediction of survival in diffuse large b cell lymphoma treated with chemotherapy plus rituximab](http://www.ncbi.nlm.nih.gov/geo/query/acc.cgi?acc=GSE10846) | diffuse large b cell lymphoma | 222444_AT | 0.00327 | **Positive** |
| [a gene signature predicting for survival in suboptimally debulked patients with ovarian cancer](http://www.ncbi.nlm.nih.gov/geo/query/acc.cgi?acc=GSE26712) | ovarian cancer | 217858_S_AT | 0.0308 | **Negative** |
| [gene expression data for pathological stage i-ii lung adenocarcinomas](http://www.ncbi.nlm.nih.gov/geo/query/acc.cgi?acc=GSE31210) | lung cancer | 222444_AT | 0.0379 | **Positive** |
| [experimentally derived metastasis gene expression profile predicts recurrence and death in colon cancer patients](http://www.ncbi.nlm.nih.gov/geo/query/acc.cgi?acc=GSE17538) | colon cancer | 222444_AT | 0.046 | **Negative** |
| [human lung adenocarcinoma](http://www.ncbi.nlm.nih.gov/geo/query/acc.cgi?acc=GSE36471) | lung cancer | 10456 | 0.0488 | **Positive** |
| [183 breast tumors from the helsinki univerisity central hospital with survival information](http://www.ncbi.nlm.nih.gov/geo/query/acc.cgi?acc=GSE24450) | breast cancer | ILMN_2334760 | 0.056 | **Positive** |
| [prediction of progression-free survival in patients with advanced-stage serous ovarian cancer](http://www.ncbi.nlm.nih.gov/geo/query/acc.cgi?acc=GSE17260) | ovarian cancer | A_24_P23411 | 0.0565 | **Negative** |
| [molecular subclasses of high-grade glioma: prognosis, disease progression, and neurogenesis](http://www.ncbi.nlm.nih.gov/geo/query/acc.cgi?acc=GSE4271) | high-grade glioma | 217858_S_AT | 0.067 | **Positive** |
| [metastasis gene expression profile predicts recurrence and death in colon cancer patients (moffitt samples)](http://www.ncbi.nlm.nih.gov/geo/query/acc.cgi?acc=GSE17536) | colon cancer | 217858_S_AT | 0.0955 | **Negative** |
| [gene expression analysis of glioblastomas identifies the major molecular basis for the prognostic benefit of younger age](http://www.ncbi.nlm.nih.gov/geo/query/acc.cgi?acc=GSE13041) | glioblastoma | 217858_S_AT | 0.101 | **Positive** |

I) B2MG

| **GEO dataset** | **Cancer Type** | **GENE (Probe ID)** | **P-value** | **Effect Sign** |
| --- | --- | --- | --- | --- |
| [metastasis gene expression profile predicts recurrence and death in colon cancer patients (moffitt samples)](http://www.ncbi.nlm.nih.gov/geo/query/acc.cgi?acc=GSE17536) | colon cancer | 232311_AT | 0.00958 | **Negative** |
| [prediction of survival in diffuse large b cell lymphoma treated with chemotherapy plus rituximab](http://www.ncbi.nlm.nih.gov/geo/query/acc.cgi?acc=GSE10846) | diffuse large b cell lymphoma | 232311_AT | 0.014 | **Positive** |
| [genome-wide profiling of astrocytic gliomas](http://www.ncbi.nlm.nih.gov/geo/query/acc.cgi?acc=GSE18166) | astrocytic gliomas | 624 | 0.0159 | **Positive** |
| [a prognostic gene expression index in ovarian cancer](http://www.ncbi.nlm.nih.gov/geo/query/acc.cgi?acc=GSE14764) | ovarian cancer | 201891_S_AT | 0.0233 | **Negative** |
| [experimentally derived metastasis gene expression profile predicts recurrence and death in colon cancer patients](http://www.ncbi.nlm.nih.gov/geo/query/acc.cgi?acc=GSE17538) | colon cancer | 232311_AT | 0.0262 | **Negative** |
| [validation cohort for genomic predictor of response and survival following neoadjuvant taxane-anthracycline chemotherapy in breast cancer](http://www.ncbi.nlm.nih.gov/geo/query/acc.cgi?acc=GSE25065) | breast cancer | 201891_S_AT | 0.0434 | **Negative** |
| [an eight-gene expression signature for the prediction of survival and time to treatment in chronic lymphocytic leukemia](http://www.ncbi.nlm.nih.gov/geo/query/acc.cgi?acc=GSE22762) | chronic lymphocytic leukemia | 201891_S_AT | 0.0494 | **Positive** |
| [an expression signature for p53 in breast cancer predicts mutation status, transcriptional effects, and patient survival](http://www.ncbi.nlm.nih.gov/geo/query/acc.cgi?acc=GSE3494) | breast cancer | 201891_S_AT | 0.0526 | **Positive** |
| [discovery cohort for genomic predictor of response and survival following neoadjuvant taxane-anthracycline chemotherapy in breast cancer](http://www.ncbi.nlm.nih.gov/geo/query/acc.cgi?acc=GSE25055) | breast cancer | 201891_S_AT | 0.0728 | **Negative** |
| [gene expression signatures for predicting prognosis of squamous cell lung carcinomas](http://www.ncbi.nlm.nih.gov/geo/query/acc.cgi?acc=GSE4573) | lung cancer | 216231_S_AT | 0.0782 | **Positive** |
| [expression data for early stage nsclc](http://www.ncbi.nlm.nih.gov/geo/query/acc.cgi?acc=GSE19188) | lung cancer | 232311_AT | 0.0805 | **Negative** |
| [relapse-related molecular signature in lung adenocarcinomas identifies patients with dismal prognosis](http://www.ncbi.nlm.nih.gov/geo/query/acc.cgi?acc=GSE13213) | lung cancer | A_23_P37441 | 0.0821 | **Positive** |
| [strong time dependence of the 76-gene prognostic signature](http://www.ncbi.nlm.nih.gov/geo/query/acc.cgi?acc=GSE7390) | breast cancer | 201891_S_AT | 0.104 | **Negative** |

**Supplementary Table 2.**

A)

| **Gene name** | **GEO dataset** | **Cancer Type** | **P-value** | **Effect of high expression on survival** |
| --- | --- | --- | --- | --- |
| VPS26A | [183 breast tumors from the helsinki univerisity central hospital with survival information](http://www.ncbi.nlm.nih.gov/geo/query/acc.cgi?acc=GSE24450) | breast cancer | 0.00417 | **Positive** |
| LANCL1 | [183 breast tumors from the helsinki univerisity central hospital with survival information](http://www.ncbi.nlm.nih.gov/geo/query/acc.cgi?acc=GSE24450) | breast cancer | 0.00814 | **Positive** |
| EBP50 | [183 breast tumors from the helsinki univerisity central hospital with survival information](http://www.ncbi.nlm.nih.gov/geo/query/acc.cgi?acc=GSE24450) | breast cancer | 0.549 | **Positive** |
| NTAL | [183 breast tumors from the helsinki univerisity central hospital with survival information](http://www.ncbi.nlm.nih.gov/geo/query/acc.cgi?acc=GSE24450) | breast cancer | 0.0576 | **Positive** |
| ARMCX3 | [183 breast tumors from the helsinki univerisity central hospital with survival information](http://www.ncbi.nlm.nih.gov/geo/query/acc.cgi?acc=GSE24450) | breast cancer | 0.056 | **Positive** |
| B2M | [183 breast tumors from the helsinki univerisity central hospital with survival information](http://www.ncbi.nlm.nih.gov/geo/query/acc.cgi?acc=GSE24450) | breast cancer | 0.127 | **Positive** |

B)

| **Gene name** | **GEO dataset** | **Cancer Type** | **P-value** | **Effect of high expression on survival** |
| --- | --- | --- | --- | --- |
| LANCL1 | [validation cohort for genomic predictor of response and survival following neoadjuvant taxane-anthracycline chemotherapy in breast cancer](http://www.ncbi.nlm.nih.gov/geo/query/acc.cgi?acc=GSE25065) | breast cancer | 0.0632 | **Positive** |
| EB50 | [validation cohort for genomic predictor of response and survival following neoadjuvant taxane-anthracycline chemotherapy in breast cancer](http://www.ncbi.nlm.nih.gov/geo/query/acc.cgi?acc=GSE25065) | breast cancer | 1.5e-05 | **Positive** |
| STX4 | [validation cohort for genomic predictor of response and survival following neoadjuvant taxane-anthracycline chemotherapy in breast cancer](http://www.ncbi.nlm.nih.gov/geo/query/acc.cgi?acc=GSE25065) | breast cancer | 0.0135 | **Positive** |
| VAMP3 | [validation cohort for genomic predictor of response and survival following neoadjuvant taxane-anthracycline chemotherapy in breast cancer](http://www.ncbi.nlm.nih.gov/geo/query/acc.cgi?acc=GSE25065) | breast cancer | 0.0999 | **Positive** |

**Supplementary Table 3. Antibodies used in the study.**

**A)** Western Blot

| **Name** | **Source** | **Dilution** | **Molecular Weight** | **Supplier** |
| --- | --- | --- | --- | --- |
| Actin | Mouse | 1:10,000 | 42 kDa | Abcam |
| Calnexin | Rabbit | 1:1000 | 95KD | Cell Signalling #2433 |
| Na/K ATPase | Mouse | 1:4000 | 100 kDa | Ab7671 |
| STX4 | Mouse | 1:1000 | 33 KDa | Ab77037 |
| MAPK | Mouse | 1:1000 | 120 kDa | Cell Signalling #9102S |
| p21 | Mouse | 1:300 | 21 kDa | Santa Cruz (SX118): sc-53870 |
| p53 | Mouse | 1:1000 | 53KD | Santa Cruz: sc-126 |
| NOTCH3 | Rabbit | 1:1000 | 90- 270KD | Cell Signalling #5276 |
| DEP1 | Mouse | 1:1000 | 220 kDa | Santa Cruz: sc-376749 |
| DCR2 | Rabbit | 1:1000 | 40 kDa | Abcam[EPR3588(2)] |
| NTAL | Mouse | 1:1000 | 33KD | Ab3992 |
| EBP50 | Rabbit | 1:500 | 50KD | Ab3452 |
| ARMCX3 | Rabbit | 1:500 | 36 | Ab98938 |
| PLD3 | Rabbit | 1:1000 | 55 | Novus Biologicals (NBP1-59921) |
| VAMP3 | Rabbit | 1:200 | 13 | Ab68833 |
| LANCL1 | Rabbit | 1:500 | 40 | Santa Cruz (H-45): sc-134677 |
| p16 | Mouse | 1:1000 | 16 | Ab54210 |
| B2M | Mouse | 1:100 | 13 | Ab759 |
| VPS26 | Rabbit | 1:1000 | 40 | Ab23892 |

**B)** Immunofluorescence

| **Name** | **Source** | **Dilution** | **Molecular Weight** | **Supplier** |
| --- | --- | --- | --- | --- |
| STX4 | Mouse | 1:200 | 33 KDa | Ab77037 |
| DEP1 | Mouse | 1:100 | 220 kDa | Santa Cruz: sc-376749 |
| DCR2 | Rabbit | 1:100 | 40 kDa | Abcam[EPR3588(2)] |
| NTAL | Mouse | 1:200 | 33KD | Ab3992 |
| EBP50 | Rabbit | 1:200 | 50KD | Ab3452 |
| ARMCX3 | Rabbit | 1:100 | 36 | Ab98938 |
| VAMP3 | Rabbit | 1:100 | 13 | Ab68833 |
| B2M | Mouse | 1:100 | 13 | Ab759 |

**C) Immunohistochemistry**

| **Name** | **Source** | **Dilution** | **Supplier** | **Reacts with** |
| --- | --- | --- | --- | --- |
| p16^INK4a^ | Rabbit | 1:100 | Santa Cruz SC-1207 | M |
| p19^ARF^ | Rabbit | 1:100 | Abcam[EPR3588(2)] | M |
| NTAL | Mouse | 1:100 | Ab3992 | H/M |
| STX4 | Mouse | 1:100 | abcam77037 | H/M |
| DEP1 (PTPRJ) | Rabbit | 1:100 | Bioss bs-2567R | M |
| B2M | Mouse | 1:100 | Ab759 | H |

**D)** Flow cytometry

| **Name** | **Dilution** | **Supplier** | **Tagged with** |
| --- | --- | --- | --- |
| DEP1 | 1:5 | R&D Systems, FAB1934P | PE |
| B2MG | 1:5 | NOVUS BIOLOGICALS, NB100-77981 | FITC |
| DCR2 | 1:5 | R&D Systems, FAB633G | FITC |
| NOTCH3 | 1:5 | eBIOSCIENCE, 17-5787 | APC |
